# Effect of Hydroxyapatite Coating in Combination with Physical Modifications on Microshear Bond Strength of Zirconia to Resin Cement

**DOI:** 10.1155/2023/9523683

**Published:** 2023-01-10

**Authors:** Faezeh Atri, Vanya Rasaie, Sakineh Nikzad Jamnani, Saba Mohammadi

**Affiliations:** ^1^Department of Prosthodontics, School of Dentistry, Tehran University of Medical Sciences, Tehran, Iran; ^2^Department of Prosthodontics, Dental Research Center, Dentistry Research Institute, Tehran University of Medical Sciences, Tehran, Iran; ^3^Department of Prosthodontics, Faculty of Dentistry, Tehran University of Medical Sciences, Tehran, Iran; ^4^Faculty of Dentistry, Tehran University of Medical Sciences, Tehran, Iran

## Abstract

**Background:**

Zirconia has been used as a reliable core material in dental restorations for years; however, its bonding to resin cement is a matter of challenge. Physical, chemical, and combinations of these techniques have been investigated to boost the properties of zirconia surface bonding. The objective of this work was to evaluate the effect of hydroxyapatite coating as a chemical therapy in combination with physical modifications on the microshear bond strength of the resin cement over zirconia.

**Methods:**

In the present research, 60 sintered zirconia blocks (4 × 4 × 4 mm) were randomized into four groups of 15, including Al_2_O_3_ particle abrasion (group 1), HA coating (group 2), Al_2_O_3_ particle abrasion + HA coating (group 3), and Er, Cr: YSGG laser irradiation + HA coating (group 4). The microshear bond strength was determined by bonding the blocks to the resin cement.

**Results:**

The bond strengths (mean ± standard deviation) of modified zirconia surfaces were 16.93 ± 4.94 MPa, 16.14 ± 5.4 MPa, 19.4 ± 5.27 MPa, and 16.21 ± 3.7 MPa in groups 1–4, respectively. Test results of the ANOVA test revealed no significant difference regarding the bond strength values of zirconia surfaces to the resin cement between the studied preparation modalities (*p* > 0.05).

**Conclusion:**

Observations from the present study showed that HA coating can be as effective as the air-borne particle abrasion technique in improving bond strength to zirconia surfaces. Moreover, sandblasting by an aluminum oxide or Er, Cr: YSGG laser irradiation prior to HA coating of zirconia showed no significant effect on the reinforcement of bond strength values when compared to HA coating alone. The clinic hydroxyapatite coating alone or in combination with physical treatments improves the bond strength of zirconia to resin cement.

## 1. Background

All-ceramic materials optimized for mechanical properties have recently attracted special attention for use in the manufacture of fixed metal-free restorations. Among these, zirconium oxide (ZrO_2_) is one of the most popular ceramics [1]. This ceramic has desirable characteristics such as biocompatibility, good appearance, and chemical stability. Thus, it becomes a favorable material for various applications in dentistry, such as dental braces, posts, implant restoration abutments, and the framework of fixed partial prostheses. Likewise, it is applicable in conservative restorations, including veneers, inlays, onlays, and Maryland bridges. The nonretentive preparation of these restorations has given special importance to the bond strength between teeth and restorations [2]. More conservative tooth preparation, improved marginal adaptation, increased restoration retention, and reduced microleakage are among the advantages of a chemical bond of the restoration with the resin cement [3]. Hence, it is of clinical importance to achieve a chemical bond on the zirconia surface, especially when the macromechanical retention is compromised. Moreover, it has been shown that the coupling agents were not successful in the attainment of an acceptable chemical bond between the resin cement and zirconia disparate silica-based ceramics due to their polycrystalline glass-free structure that cannot be etched by common acids [4]. Resin-bond strengths are associated with their potential to penetrate into the surface irregularities of the underlayer [5]. The methods to achieve improved bond strength of zirconia to resin cement can be classified into 3 groups, including physical, chemical, and physicochemical techniques. Tribochemical treatment by RocatecTM is the available physicochemical technique for ceramics like Y-TZP. In this technique, silica-coated aluminum particles are subjected to low-pressure sandblasting, followed by silica-mediated chemical modification of the ceramic surface [6]. The application of primers containing organophosphate monomers and carboxylic acid is among the chemical techniques for improving adhesion [6]. Resin cement containing 4-methacryloxyethyl trimellitate anhydride (4-META), methacryloxy decyl phosphoric acid (MDP), or 3-trimethoxysilylpropyl methacrylate (MPS) monomers could have a reaction with oxide groups in the structure of Y-TZP crystalline. This process resembles the interaction between the silane coupling agent and silica-based ceramics [7]. Air-borne particle abrasion with aluminum oxide and laser etching are examples of creating surface roughness as a physical technique [8]. Alumina sandblasting can successfully enhance the surface area and create an active surface with increased wettability in dental materials [9]. Nonetheless, this method has been criticized for its adverse effects on the mechanical profiles of zirconia like flexural strength as well as the possibility to induce subcritical crack growth within the material [9]. Lasers have different applications in dental treatments. The neodymium-doped: yttrium aluminum garnet (Nd: YAG) laser can be used for reducing tooth sensitivity, caries removal, bleaching, and producing surface roughness in high-strength core ceramics [10]. Liu et al. [11] demonstrated a rougher surface of zirconia ceramics with a higher output power of the Nd: YAG laser. However, the shear bond strength of the laser group was not clearly increased. The pulsed erbium lasers (i.e., Er, Cr: YSGG, and Er: YAG) have been applied for the treatment of surfaces. Kasraei et al. [12] revealed that the bond strength of zirconia to resin cement was greater in groups treated with the Er: YAG laser in comparison to CO_2_laser-treated samples. Referring to the findings, the Er, Cr: YSGG lasers can generate comparable surface roughness rather than acid etching of dentin or enamel surfaces [13]. Likewise, it could effectively change the surface roughness of the lithium disilicate ceramics and increase the shear bond strength of this ceramic to resin cement [14]. Contradictory findings are available regarding the preference for different surface conditioning approaches to improve the adhesion of resin cement to zirconia. The inorganic matrix of bone and teeth of human beings can be found in hydroxyapatite or HA [Ca10 (PO4)6·(OH)2], as phosphocalcic hydroxyapatite [15]. It exhibits admirable biocompatibility, with a crystal structure and composition like apatite in the skeletal and dental architectures of humans. Seo et al. [16] fabricated a coating of HA zirconia substrate by a room-temperature spray process. They exhibited no severe dissolution as observed during in vitro experimentation [17]. Significant advances in nanotechnology have suggested practical applications for nano-HA in dentistry, with crystals ranging from 50 to 1000 nm. Nano-HA possesses bioactive and biocompatible properties. Moreover, they are similar to the tooth enamel apatite crystal morphologically, and in terms of crystallinity and crystal architecture [18]. Furthermore, nano-HA has a filler function because it repairs tiny depressions and holes on the surface of enamel, an action that increases with the small size of the constituent particles [15]. Nanoparticles produce a thin and consistent coating layer with adequate strength [19]. Nanosized HAp increases the level of crystallinity in glass ionomer cement and enhances mechanical features such as microshear bond strength [20]. Panavia F 2.0 is an extensively applied resin cement in dentistry that contains a bifunctional monomer, 10-methacryloyloxydecyl dihydrogen phosphate (MDP). According to the favorable adhesion of the resin cement to the tooth structure, which is mainly composed of HA, it is hypothesized that a coated layer of HA on the zirconia could result in a suitable bond strength. The present study investigated the influence of HA coating on microshear bond strength (*μ*SBS) of zirconia to resin cement (Panavia F 2.0). There were two null hypotheses: (1) there is no difference in the *μ*SBS among the study groups. (2) There is no difference in the patterns of failure.

## 2. Materials and Methods

This research is in line with the previous work that was also conducted by these researchers [21]. In the current in vitro work, 60 pre-sintered commercial dental zirconia blocks (Cercon, Dentsply, Amherst, N.Y.) with dimensions of 4 × 4 × 4 mm were ground for 60 s via 600 and 800 Struers RotoPol 11 silicon carbide grit abrasive (Struers A/S, Rodovre, Denmark), followed by cleaning in an i600B steam cleaner (Italy ELT) for 10 minutes using ethanol (96%) and subsequently air-drying for 30 s. In accordance with earlier identical work [21] and regarding the Bonferroni formula for the determination of the sample size, the number of specimens in each group was determined as *n* = 15. The sampling method was nonrandomized in this study. However, the allocation of specimens to each group was randomized.

Group 1 consisted of zirconia blocks sandblasted with Al_2_O_3_ particles (50 *μ*m) under a pressure of 4 kg/cm^2^ and a distance of 10 mm for 15 s in a MESTRA sandblast apparatus (TALLERES MESTRAITUA S. L., Espana), followed by cleaning with ethanol (96%) for 10 minutes in an ultrasonic cleaner. Group 2 consisted of zirconia blocks whose surfaces were coated via the HA thermal protocol so that a slurry solution was prepared by adding 10 gr of nanoparticle HA powder (less than 100 nm) (Merck, Germany) to distilled water (50 cc). Then, 1 gr of polyvinyl alcohol (Merck, Germany) was also added as the binder of the suspension, followed by heating on a magnet stirrer with 1000 rpm at 100°C for 60 min to achieve a uniform suspension. At last, the zirconia blocks were placed in this slurry at an angle of 45° for 5 s.

Group 3 consisted of zirconia blocks sandblasted with the same condition as described in group 1 and then layered with a coating of HA following the same procedure as described in group 2.

Group 4 consisted of zirconia blocks whose surfaces were first exposed to Er, Cr: YSGG laser irradiation with the following details: a wavelength of 2.78 *μ*m, 140 *μ*s pulse duration, a 20 Hz repetition rate, and 4 W output power. The laser optical fiber (with a diameter of 600 *μ*m and a length of 6 mm) with the gold handpiece was positioned to the surface at a distance of 10 mm perpendicularly and moved in a sweeping manner manually within a 30 s exposure time over the whole area. Continuous water (55%) and air (65%) flows were applied while performing irradiation. After laser treatment, the surface was coated with HA, as explained for group 2.

**Figure 1:**
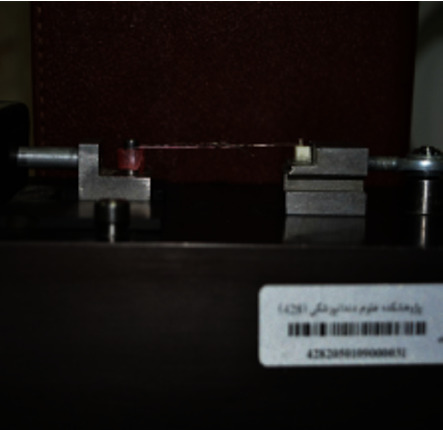
Microshear test.

Subsequent to the surface treatment, all blocks in groups 2, 3, and 4 were sintered following this protocol: ambient temperature to 300°C for 10 minutes, 300–600°C for 10 minutes, 600–900°C for 30 minutes, 900–1200°C for 40 minutes, maintenance at 1200°C for 120 minutes, and cooling for annealing, employing a CWF furnace (Keison Products, UK). Following the surface treatment in all study groups, the mold was selected to be Tygon Norton Performance Plastic tubes with an inner diameter of 0.8 mm and a height of 1 mm (Cleveland, OH, USA) for the Panavia F 2.0 cement bonding (Kuraray Medical Inc.) to the prepared surfaces. Then, the tubes were removed with a heated, sharp scalpel. All samples were placed in distilled water for 24 hours, incubated (incubator, model PL-455G, PecoPooya Electronic Co.) at 37°C, and positioned in a microtensile tester (Bisco Inc., USA) for the determination of microshear bond strength. A metal loop (0.2 mm thick; Ligature, Dentsply GAC_SOF) was put around the cement cylinder in the bonding site for the exerted tensile load conversion to shear load through vertical soldering of casting molds to the jig, as shown in Figure 1. The load extent at failure (crosshead speed = 0.5 mm/min) was determined, followed by computing the values of microshear bond strength in accordance with the equation *S* (MPa) = *F* (*N*)/*A* (mm). Following the shear experiment, an SEM was used to determine the fractured surfaces for achieving the failure variants that included cohesive failure, adhesive failure, and mixed failure.

### 2.1. Statistical Analysis Method

To analyze the data, the SPSS software version 21.0 was used. The statistical comparison of the bond strength was conducted with a one-sided analysis of variance (ANOVA) among the 4 groups. In the present study, the type 1 error rate (*α* or *p* value) was considered equal to 0.05.

## 3. Results

The mean *μ*SBS in groups 1, 2, 3, and 4 were calculated as 16.93 ± 4.94 MPa, 16.14 ± 5.4 MPa, 19.4 ± 5.27 MPa, and 16.21 ± 3.7 MPa, respectively (Table 1). According to a one-sided analysis of variance results, the differences in the amount of *μ*SBS were not significant among the study groups (*p*=0.23). Since there were no significant differences as determined by the ANOVA analysis, no pairwise comparison was performed between the groups.

**Figure 2:**
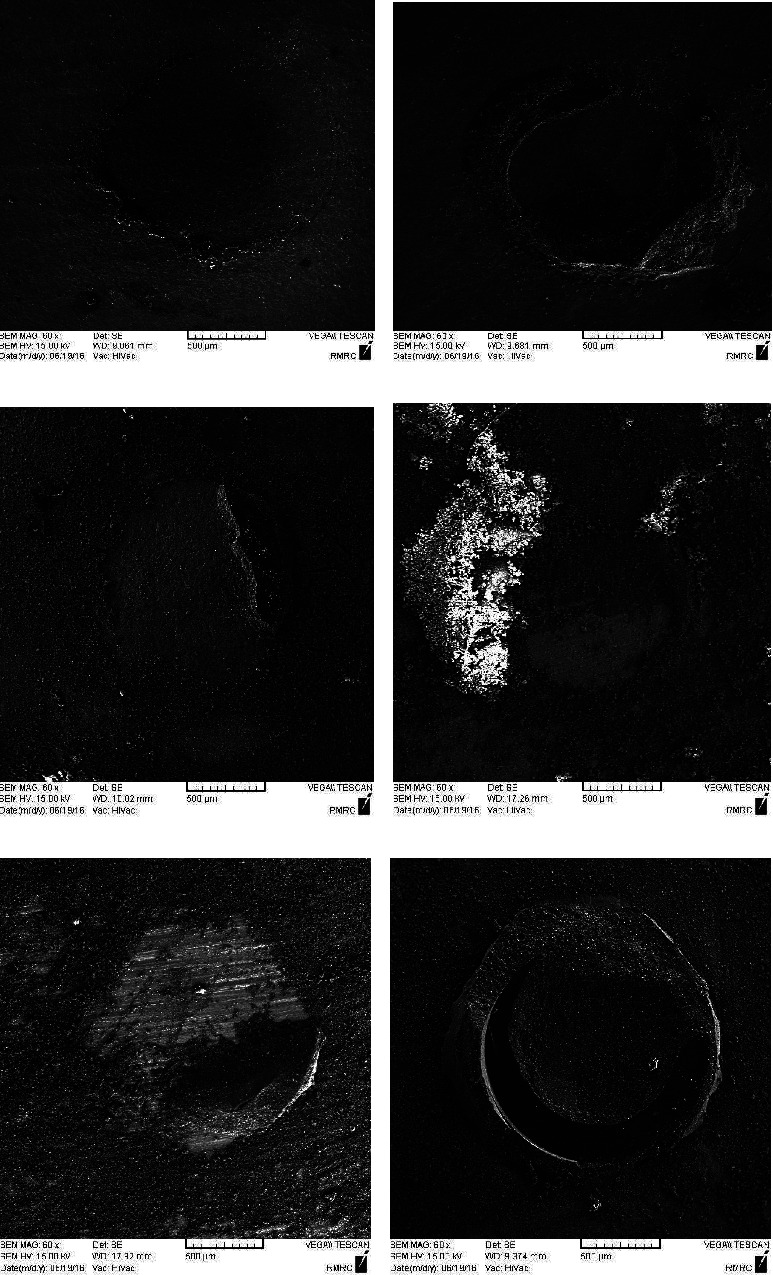
Al_2_O_3_ particle abraded group (group 1). (a) Adhesive failure mode. (b) Mixed failure mode. (c) Sandblast + HA coating group (group 3). (d) HA coated group (group 2) Er, Cr: YSGG + HA-coating group (group 4). (e) Mixed failure mode. (f) Cohesive failure mode.

The most common failure modes in all study groups were the adhesive types. All cohesive fractures occurred in the cement cylinder. The mixed type of failure was the less common type of fracture in groups 1 and 3.  Table 2 describes the number of different types of fractures in the study groups (Figure 2).

## 4. Discussion

According to the mineral tooth structure, which is mainly composed of hydroxyapatite crystals, and the favorable resin cement bond strength to such a structure, it is hypothesized that coating the zirconia surface with the nanosized HA might boost the bond strength with resin cement. The current effort investigates the influence of hydroxyapatite coatings solely and in combination with physical surface modification via Er, Cr: YSGG laser irradiation, and air-borne particle abrasion. In our research line, Azari et al. [21] were just the pilot study for the surface treatment of zirconia with hydroxyapatite. In the following, Azari et al. [19] compared the bonding strength of zirconia to cement resin in two techniques: HA coating and air abrasion. In the present study, we tested the effect of HA coating in combination with physical surface treatments such as air abrasion and laser on the bond strength of resin cement. The results indicated that the null hypothesis should be accepted, as the *μ*SBS was not significantly different among the study groups. While Y-TZP offers many advantages, such as high strength, fracture toughness, and good wear resistance, there are some disadvantages, such as the nonpolar nature, which leads to negligible bonding capacity with dental structure and/or overlaying ceramics [22]. Multiple chemical and mechanical methods have been reportedly combined for the enhancement of the bonding activity of dental zirconia [1]. The surface of zirconia can be modified by a HA coating, which has been previously used as a promising method to modify bioinert metallic surfaces in implant dentistry [16]. As reported by Sagsoz et al., the highest bond strength within resin ceramics and resin cement was among the HAp group. According to their conclusions, HAp coating can be used instead of hydrofluoric acid etching and sandblasting in CAD/CAM materials and resin cement to improve the bond strength [23].

Over the years, using digital technologies in dentistry has developed. In a study by Pagano et al. [24], CAD/CAM prostheses had improved mechanical characteristics in frequency scopes. Besides, they are less cytotoxic than conventional prostheses. Also, a novelty in prosthetics is the use of milled discs of polymethylmethacrylate (PMMA) with CAD/CAM machinery, which exhibit adequate mechanical features and greater biocompatibility especially for removable prostheses. In addition, Brillouin spectroscopy and dynamic mechanical analysis (DMA) are pioneering procedures for mechanical analysis [24].

There are diverse coating approaches available for exploiting the merits of HA. No standard instructions have been issued so far for the various dimensions of a particular approach, such as surface profile, porosity, texture, coating thickness, and crystallinity or crystal size effects. Several methods of coating a HA layer onto bioinert metallic implant surfaces have been previously reported, which include the plasma spray and sol-gel methods. The present work benefited from the thermal coating approach because of its fine mechanical profiles, practical feasibility, and admirable phase composition [21]. The thermal coating technique has possibilities for modifying the surface features of zirconia and might be capable of shifting the bonding pattern of zirconia ceramics [21]. As concluded by Okada et al., the thermal coating of zirconia after applying silane coupling agents has the capacity to boost the shear bond strength within the zirconia and composite resin cement [25]. Previous research has indicated that, in comparison to the control group, air-borne particle abrasion significantly boosted the bond strength of the zirconia surface to Panavia F 2.0 resin cement [21]. In addition, one of the best surface treatment approaches is sandblasting for surface modification of zirconia ceramics [26]. Thus, it was employed as the control group in our work. Controversies and limitations of the sandblasting method can be addressed by coating the zirconia surface via HA regarding its merits, some of which are great surface tension, radiopacity, optimal hardness for natural teeth, a potent wear profile, and admirable bonding and wettability. The present study revealed that the HA-decorated zirconia surface boosted the bond strength to resin cement similar to the sandblasted group. However, the combination of other surface treatments such as Er, Cr: YSGG laser irradiation, or air abrasion has shown no additional benefit in the improvement of bond strength. The lowest range for acceptable clinical bonding was suggested to be between 10 and 13 MPa [27]. The *μ*SBS values of all study groups were beyond the acceptable range, and their differences were not statistically significant. As stated by Ranjbar Omidi et al., silica coating through Cojet sand presented substantially greater *μ*-shear bond strength compared to Er: YAG laser and sandblasted groups. Moreover, the *μ*SBS values of the sandblasted group were higher than those of the Er: YAG laser [28]. Sandblasting increases the bond strength between zirconia and resin cement [21]. Tanış and Akçaboy [29] indicated that the values of shear bond strength between Y-TZP and Panavia F 2.0 were significantly greater in the group modified with sandblasting + tribochemical silica coating + silane in comparison to the sandblasted specimens. Consistent with this, Zandparsa et al. [26] reported that the sandblasted zirconia surface showed inferior shear bond strength to enamel in comparison to the Al_2_O_3_ air abraded + Z-PRIME Plus group. According to the findings of these two studies and other similar studies [1], it might be assumed that an increase in surface roughness via laser irradiation or air-borne particle abrasion in combination with modifying the surface with a layer of HA might result in higher values of *μ*SBS. Although the *μ*SBS value was higher in group 3 in comparison to group 1, such a difference was not statistically significant. This might be due to the replacement of the surface irregularities with nanosized HA, which would leave no efficient indentation for micromechanical retention of the cement layer. We coated the surface of zirconia with nano-HA on the basis of a thermal protocol. Nanoparticles provided a thin and uniform controllable coating layer with adequate strength. In addition, Abdulkader and Aljubori reported that the addition of nanohydroxyapatite (nHAp) to self-adhesive resin cement reinforced the mechanical features and bond strength of zirconia [30, 31]. There is limited scientific evidence compared to the amount of surface roughness produced via Er, Cr: YSGG laser irradiation, and Al_2_O_3_ particle abrasion. An SEM observation revealed more surface irregularities with 50 *μ*m Al_2_O_3_ abrasion than the Er, Cr: YSGG laser (2 W and 3 W) treated Y-TZP surface [27]. Although specific surface roughness tests are required for more accurate conclusions on differences in surface roughness, the higher values of *μ*SBS in group 3 of the present study, in comparison to group 4, could be the result of better micromechanical bonds. While several studies have investigated the impact of Al_2_O_3_ particle abrasion and its influencing factors on the sintered zirconia surface roughness [32]. In the present study, particle size was utilized. The data of the previous studies reported that the 50 *μ*m Al_2_O_3_ particle abrasion could result in elevated flexural strength because of the formation of compressive fields owing to the induced tetragonal–monoclinic transformation of the crystals present in the surface [33]. However, such transformation and the formation of small flaws might compromise the fatigue strength of Y-TZP restoration in the long term [34]. In a work by Kern and Wegner [35], the phosphate ester monomer of MDP exhibits a water-resistant durable chemical bonding. The MDP monomer, with bonding agents, reportedly could boost the bond strength of sandblasted zirconia with resin cement [36]. Hence, we recruited Panavia F 2.0 in our experiments. The SEM images captured to analyze the failure mode revealed that the majority of fractures were adhesive in the solely air-abraded group (group 1), and other groups coated with HA showed more mixed fractures than group 1. It can be related to the high quality of the chemical bond of resin cement with coated HAp zirconia [19]. The present paper is a continuation of previous work on the improved bonding characteristics of zirconia ceramics [27, 29]. Similarly, Azari et al. concluded that failure patterns in the HAp-coated group were mixed and adhesive in the sandblasting and control [19]. SEM observations also revealed some of the adhesive failures in the Er, Cr: YSGG groups. While it seems that HA coatings can be considered for the preparation of zirconia surfaces before using luting cement, the effect of temperature, moisture environment, and cyclic loads is still unclear for this mode of surface modification. In addition to more in vitro evidence on the applicability of this technique, further clinical investigations are required in this regard. In line with the beneficial adhesion of the resin cement to the tooth structure, the influence of the HA coating on the bond strength of zirconia to resin cement (Panavia F 2.0) was evaluated in this study. Lately, a new material named Activa™ Bio-Active Restorative has been presented. As highlighted by the producer, a feature of bioactive substances is the probable appearance of HA at the point of contact within the material and dental tissue. This material has the characteristics and benefits of both glass ionomer cement and resin composites. It also has the chemical and physical features of natural teeth. In future studies, the use of this material as a frontier in the science of dental materials is recommended [37].

The main limitation of this research was achieving a uniform HA coating on the zirconia. It was a technique-sensitive procedure. The most common test methods used to evaluate adhesive bonding between two materials are tensile, microtensile, and shear bond strength tests [38]. The shear bond strength test has an easy application procedure because no additional process is required once the bonding procedure is complete. But this test method has the disadvantage of inhomogenous stress distribution [29, 38]. One limitation of this study is that the shear bond strength test was used because of the easy application procedure. However, the microshear test shows a lower standard deviation than the shear test because the small adhesive interface used in the microshear test contains fewer defects compared to the larger specimens used in the shear test [29].

## 5. Conclusion

The following conclusions were obtained in the present work:

- The *μ*SBS of zirconia were improved by HA coating surface treatment
- Improvement of *μ*SBS with HA surface treatment is comparable to air-borne particle abrasion
- To improve the bond strength, there is no significant additional benefit from combining air-borne particle abrasion or laser irradiation with HA coating
- HA coating might be a promising alternative method for air-borne particle abrasion on zirconia surfaces

## Figures and Tables

**Table 1:** Central dispersion indices of *μ*SBS of resin cement on zirconia surface in different preparation groups.

Groups	Mean (MPa)	Standard deviation	Minimum	Maximum
(1) Sandblasting with Al_2_O_3_	16.93	4.9	10.3	26.4
(2) HA coating	16.14	5.4	7.9	26
(3) Sandblasting + HA coating	19.4	5.2	9.5	28.6
(4) Er, Cr: YSGG + HA coating	16.21	3.7	10.9	23.6

**Table 2:** Dispersion and type of failure mode among the 4 study groups.

Groups	Adhesive	Cohesive	Mixed
(1) Sandblasting with Al_2_O_3_	11 (73.3%)	1 (6.7%)	3 (20%)
(2) HA coating	8 (53.3%)	2 (13.3%)	5 (33.3%)
(3) Sandblasting + HA coating	9 (60.0%)	3 (20.0%)	3 (20.0%)
(4) Er, Cr: YSGG + HA coating	8 (53.3%)	2 (13.3%)	5 (33.3%)

## Data Availability

All the data generated or analyzed during this study are included within the article.
